# Supplementary material for: Dynamic redox and nutrient cycling response to climate forcing in the Mesoproterozoic ocean
Source: Nat Commun. 2023 Oct 20;14:6640. doi: 10.1038/s41467-023-41901-7 (PMC10589307; doi:10.1038/s41467-023-41901-7)
Supplement: Supplementary file 1 — Supplementary Information [file 41467_2023_41901_MOESM1_ESM.pdf]

Supplementary Information for

Dynamic redox and nutrient cycling response to climate forcing in the Mesoproterozoic ocean

Yafang Song, Fred T. Bowyer, Benjamin J.W. Mills, Andrew S. Merdith, Paul B. Wignall, Jeff Peakall, Shuichang Zhang, Xiaomei Wang, Huajian Wang, Donald E. Canfield, Graham A. Shields, Simon W. Poulton

Geological setting and samples

The Xiamaling Formation forms part of a thick Paleo-Mesoproterozoic sedimentary succession deposited on the North China Craton (NCC)[1,2]. High-precision zircon dating of a tuff layer near the middle part of the formation, and a bentonite layer 52 m below the tuff layer, gave ages of 1384.4 ± 1.4 Ma and 1392.2 ± 1.0 Ma, respectively[3]. Therefore, deposition of the Xiamaling Formation is constrained to ~1.4 Ga, with an estimated average sedimentation rate of ~6.67 m/Myr. The Xiamaling Formation is suggested to have been deposited in a deep and quiet marine setting with water depth near or well below storm wave base[3,4].

The Xiamaling Formation in the Xiahuayuan area is divided into six units based on sedimentological and geochemical criteria, from unit 6 to unit 1 moving up-section. Detailed descriptions of the sedimentology and drilling locations of the Xiamaling Formation core are available in Wang et al.[4]. In this study, two sections of well-preserved, fine-grained siliciclastic rocks were collected and subsequently sampled at cm-scale from drill core 1 and drill core 2, stratigraphically corresponding to the upper (unit 1) and lower (unit 4) units of the Xiamaling Formation, respectively. Section A from unit 1 is 16 cm in length (stratigraphic depth: 40.80–40.96 m), and comprises persistent black shales (Fig. S1A). Section B from unit 4 is ~70 cm in length (stratigraphic depth: 331.40–332.10

m), and consists of alternating red and green mudstones, with sometimes frequent, grey-green silty layers (Fig. S1B, C). Our focus on these relatively small intervals allows us to perform multiple, very high-resolution geochemical analyses across two complete cycles in each case. This approach builds upon reported evidence for orbital cyclicity through unit 3 of the Xiamaling Formation, based largely on major element data[3], and on lower resolution analyses of redox conditions through the Xiamaling Formation[4]. Thus, our approach, which additionally incorporates novel biogeochemical modelling in relation to palaeogeography and climate forcing, provides the first detailed evaluation of both the controls on apparent redox cyclicity across individual cycles, and implications for nutrient cycling.

The silty sediments in unit 4 range from mm to cm in thickness and consist of graded and laminated beds that are characteristic of turbidites (Fig. S2). The silty turbidites, with their parallel nature, lateral persistence on the scale of outcrops (Fig. S1B; Fig. S2C-F), and the presence of thick mudstone beds between them, are typical of a distal basin-plain environment[5]. In areas above and below Section B there are occasional thicker sandy turbidites that show decimeter-wavelength hummock-like bedforms with low-angle cross-lamination (Fig. S2B), reflecting interaction of the flows with some topography on the basin floor[6,7]. Turbidites in basin-plain environments are thought to be the product of fast moving, disintegrating large-landslides that transform to low density turbidity currents and produce aerially extensive, thin sheets consisting of thin silt/sand laminae capped by mud – the D and E divisions of Bouma's classic turbidite sequence[8,9]. Analysis of such basin-plain successions shows that turbidites exhibit temporally random Poisson distributions, potentially relating to processes such as earthquakes, which also display Poisson like distributions[9]. This environmental interpretation is consistent with earlier work, indicating that the water depth was well below storm wave base[4].

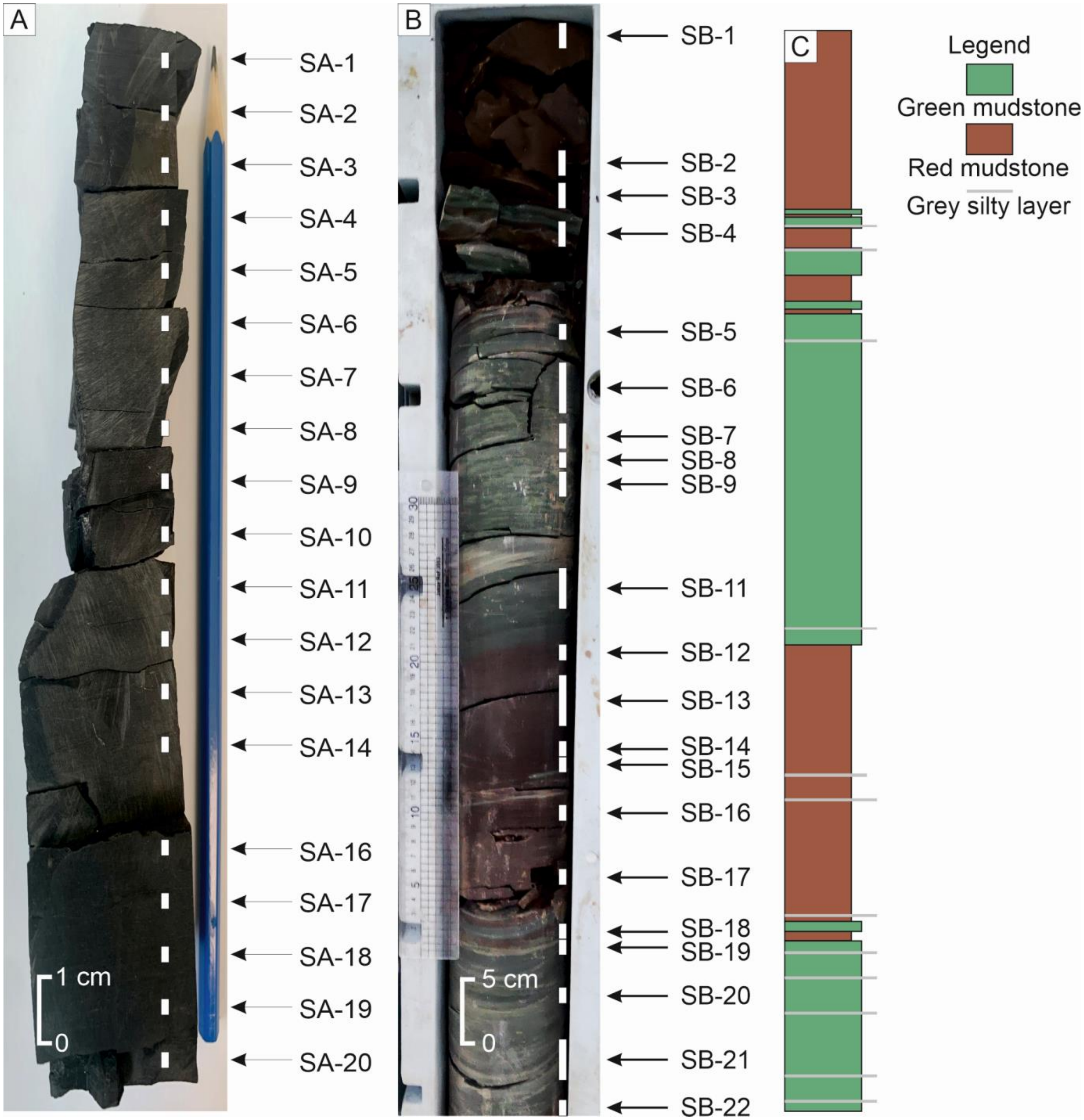

Fig. S1. Sample horizons for Section A and Section B. (**A**) Section A is composed of continuous black shales. (**B**) Section B is composed of alternating green and red mudstones, with occasional mm-scale silty layers in both the red and green muds, reflecting small-scale turbidites (shown as sedimentary log in C).

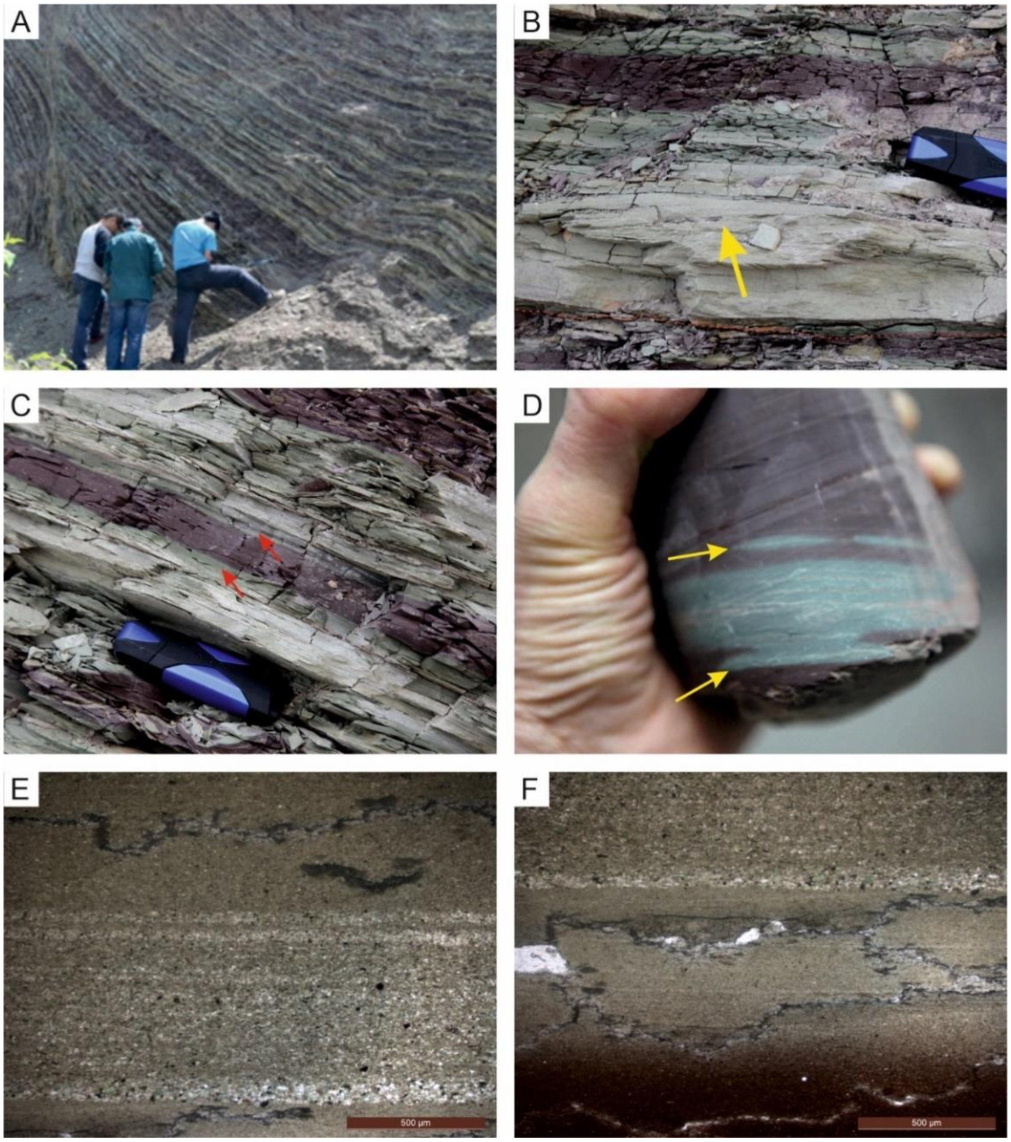

Fig. S2. Alternating red and green muds with turbidites in unit 4. (**A**) Large-scale alternating red and green muds in outcrop. (**B**) Thick (4–5 cm) turbidite (cross lamination, yellow arrow) underlain by a mm-thick green mud. (**C**) Alternating green and red muds, where turbidites are interpreted to have reduced background muds into green muds near the bottom and top of the central red mud interval (red arrows). (**D**) Lens-like green muds around thin silts (yellow arrows), likely indicating diagenetic alteration on a mm-scale. (**E**) Photomicrograph showing a graded and laminated silty turbidite interbedded with green muds (sample SB-4; see Fig. S1B). (**F**) Another level from sample SB-4, showing the development of a green reduced layer of ~1 mm thickness below the turbidite horizon, followed by downward gradation into red mudstone. Photos A-C are from Wang et al.[4] with permission.

It has been previously suggested that turbidite deposition altered the color of interbedded mudstones from red to green, due to iron reduction stimulated by remineralization of organic C transported by the turbidites[4]. Such a situation can be seen in Figure S2C, where turbidites below the central red mud layer have apparently reduced the bottom portion of the red muds. Furthermore, at the top of this red layer, a series of small turbidites have apparently reduced the background red sedimentation to a green color, while turbidites mixed with green background muds form a 3–4 cm mostly green layer. There is some residual red coloration within this mostly green layer, so it is unclear if the background muds were originally red or green in coloration. Apparent color bleed is also evident from the central turbidite into the underlying red sediment (Fig. S2B).

Thus, the color of the original deposited fine-grained sediment is difficult to ascertain in much of unit 4 due to the influence of turbidites. However, in our Section B, we observe the deposition of both red and green background muds, as there are green muds deposited without large or frequent turbidite contribution. Furthermore, thin siltstone turbidites are present in both red and green mud intervals (Fig. S1B, C). Thin turbidites, as evidenced in Section B, should only have had a small impact on the background sedimentation over distances of 1 mm or so (e.g., Fig. S2F). Thus, the thin turbidites depositing in Section B cannot be responsible for the larger scale color changes that are observed in our samples. This is in contrast to larger turbidites, discussed above, that likely had a more significant impact on the color of the rock. Thus, Section B of unit 4 is impacted by both primary color differences in the sediments, related to the original depositional environment caused by oscillations in the deeper limit of the OMZ, and the minor influence of smaller-scale turbidite deposition.

In addition to the larger-scale changes between red and green muds, there are, however, discrete finer-scale green and red mudstone alternations that commonly occur at the transition between larger-scale red and green intervals, and which are generally not influenced by turbidites (as evident at the top

of green mudstone intervals in Fig. S1B, C). We interpret these smaller scale fluctuations to reflect periodic switches in the deeper limit of the OMZ during the general progression from a more contracted to more expanded (and vice versa) OMZ.

Age model

Although low in precision, an approximate temporal framework for the two high-resolution Xiamaling sections can be constructed. On a longer timescale, sections A and B were separated by ~290 m, corresponding to a time gap of ~44 Myr based on the radiometric dates outlined above and assuming a constant sedimentation rate. On shorter timescales, the geochemistry of both sections varies at the scale of a few cm, corresponding to kyrs, which likely represents Milankovitch forcing[3].

We evaluate these shorter-term geochemical cycles by inputting pertinent aspects of our data into time-series analysis software developed by Li et al.[10]. Geochemical data that appear to show an influence from climate forcing (e.g., TOC and Mo_{EF} data from Section A; TOC and Fe_{HR}/Fe_T data from Section B) were firstly interpolated in the software to obtain a linearly spaced sampling interval. We then used Gaussian band-pass filtering to isolate potential climate forcing frequencies (Fig. S3). Passbands of 13 ± 3 cycles/m for TOC and 6.5 ± 3.5 cycles/m for Mo_{EF} were applied in Section A, yielding periodicities of 8 cm and 13 cm, respectively (Fig. S3A, B). TOC and Fe_{HR}/Fe_T data for Section B were both filtered between 2 cycles/m and 3 cycles/m passband, yielding periodicities of 33 cm and 45 cm, respectively (Fig. S3C, D). Applying the average sedimentation rate would give periodicities in Section A (8–13 cm in length) of 12–19 kyr, and periodicities in Section B (33–45 cm in length) of 49-67 kyr. However, since this estimation relies on the assumption of a constant sedimentation rate, which is of course unlikely for such an extended depositional interval, we refrain from assigning a particular orbital frequency to these intervals, since our main conclusions are unaffected by the precise nature of the orbital cyclicity.

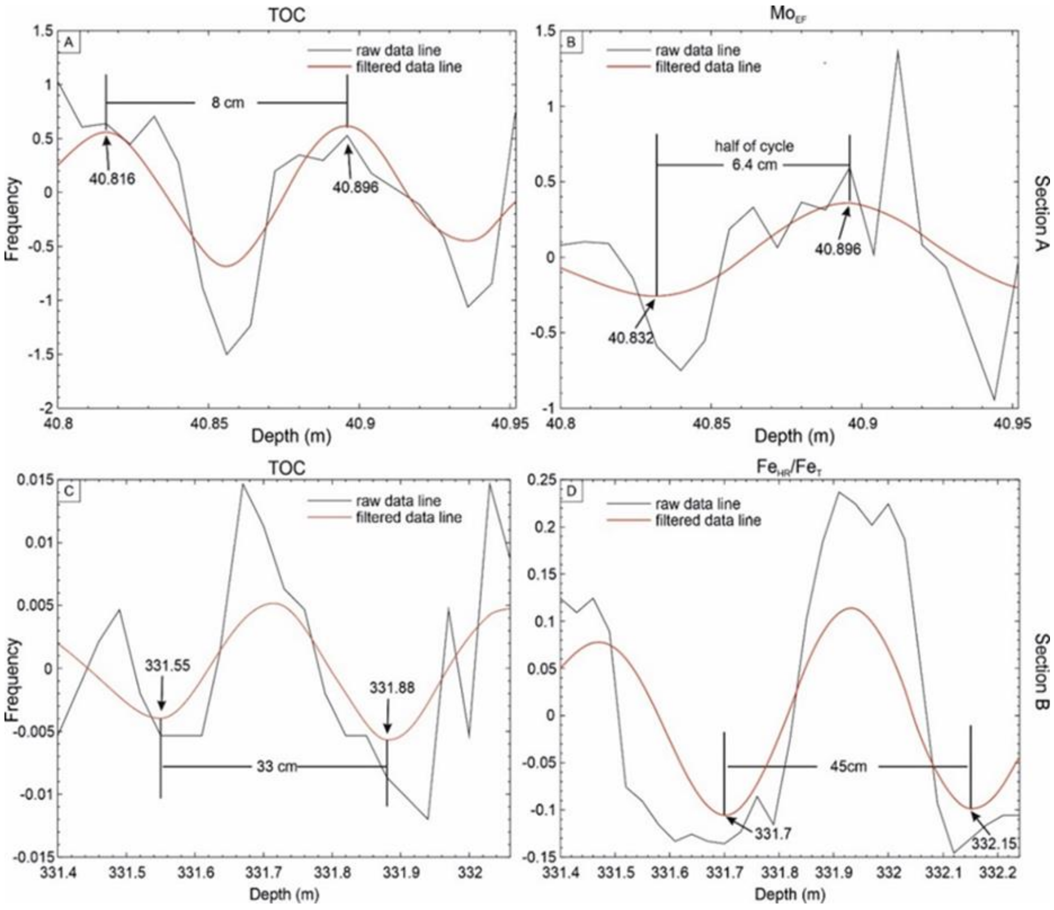

Fig. S3. Gaussian filtered geochemical periodicities. (**A**) TOC frequency shown with Gaussian filter output (red) for Section A (passbands: 13 ± 3 cycles/m). (**B**) Mo_{EF} frequency shown with Gaussian filter output (red) for Section A (passbands: 6.5 ± 3.5 cycles/m). (**C**) TOC frequency shown with Gaussian filter output (red) for Section B. (**D**) Fe_{HR}/Fe_{T} frequency shown with Gaussian filter output (red) for Section B. Passbands of 2.5 ± 0.5 cycles/m in C and D.

Paleogeographic reconstruction and climatic dynamics

We follow recent compilations of robust paleomagnetic data from NCC to help constrain its position at ~1.4 Ga[11]. Although there is no direct data available for this time, two Mesoproterozoic paleomagnetic poles (one from the ~1.44 Ga Tieling Formation, and the other from ~1.35 Ga Yanliao Mafic Sills), both indicate that the NCC was located at ~15°N[12,13]. While not prohibitive of large-scale motion, the simplest interpretation of these paleomagnetic data is that the NCC remained at equatorial to low latitudes (0–

30°N) throughout this time period. The Tieling pole is reconcilable with placing the NCC outboard of northern Australia and Siberia, with it forming part of Nuna[12,14-16]. Breakup of the Australian–Laurentian margin of Nuna from ~1.4 Ga indicates that some relative motion must have occurred between NCC, Australia and Laurentia[15], before the preservation of the Yanliao pole. However, the exact nature and magnitude of this motion is poorly constrained because of the absence of reliable palaeomagnetic data. In Figure 4A, our paleogeographic reconstruction restores Laurentia to a paleolatitude consistent with two robust Laurentian poles dated around 1.4 Ga[17]. We keep Australia and Siberia attached to Laurentia, following[11,12]. We also decouple NCC from its position at 1.4 Ga outboard of western Australia to keep it at a paleolatitude consistent with its 1.44 and 1.35 Ga paleomagnetic data (i.e., at around 15–20°N—if it remained fixed to Australia it would be between 25–30°N). The Tieling and Yanliao poles have an A_{95} uncertainty of ~6° and we use more transparent outlines of NCC to represent this uncertainty, to give an indication of permissible variability in paleolatitude (Fig. 4A). It is noteworthy that paleolongitude is not constrained.

The ~15°N location of the NCC at ~1.4 Ga likely implies a dynamic depositional environment due to its sensitive position relative to atmospheric circulation cells. The ITCZ, a tropical belt with the strongest precipitation, is widely considered to be closely linked to global climate change[18,19]. Observations from modern and ancient sediments suggest that the spatial range of the ITCZ fluctuates periodically on both seasonal and orbital timescales, controlling the dynamics of the depositional environment and thus geochemical variations[20-22]. Periodic fluctuations in sedimentology and geochemistry have been observed in the Xiamaling Formation, which are suggested to have been regulated by precipitation, trade wind and ocean circulation in response to its location relative to the ITCZ[3,4].

Paleoredox proxy systematics

Fe speciation is commonly used to distinguish three major redox conditions in ancient oceans – oxygenated, ferruginous (anoxic, iron-containing) and euxinic (anoxic, sulfidic)[23,24]. Samples deposited from an anoxic water column commonly have elevated highly reactive iron (Fe_{HR}) to total iron (Fe_T) ratios ($Fe_{HR}/Fe_T > 0.38$), while samples with $Fe_{HR}/Fe_T < 0.22$ are generally considered to provide an indication of oxic water column conditions, with Fe_{HR}/Fe_T ratios between 0.22 and 0.38 considered equivocal[23]. For anoxic samples, the extent of pyritization (Fe_{py}) of Fe_{HR} is used to indicate whether samples were deposited from a ferruginous water column ($Fe_{py}/Fe_{HR} < 0.6$), or a euxinic water column ($Fe_{py}/Fe_{HR} > 0.8$), with ratios between 0.6-0.8 considered equivocal[24].

A recent study has argued that Fe speciation data may be compromised by diagenetic processes[25]. Indeed, a number of considerations must be evaluated when applying the method. For example, Fe speciation may not always provide reliable results when applied to sediments with low Fe contents (Fe_T <0.5 wt%)[26], sediments experiencing rapid deposition (e.g., turbidites, which we note are very minor in our samples and hence would not significantly affect the background geochemical data; Fig. S1)[27], and those in proximity to hydrothermal inputs[28] or directly adjacent to (sub)tropical mountainous regions, where highly weathered sediment may supply a high proportion of Fe_{HR} directly onto the continental margin[29], thus circumventing the preferential trapping of Fe_{HR} that usually occurs in inner shore environments[30]. However, these caveats have been well described in the literature, but were ignored in the analysis of Pasquier et al.[25]. Furthermore, Fe speciation has been extensively calibrated using ancient rocks[26, 30-32], which by definition have undergone the diagenetic modification that Pasquier et al.[25] claim compromises the use of the proxy as a paleoredox tool. As also described in the literature, Fe speciation is best used in combination with other indications of water column redox chemistry (e.g., redox sensitive trace metal systematics) and within the context of the sedimentological conditions of the depositional

environment[24]. This approach has increasingly been used[33-35], and is done here to provide the most robust assessment of the chemical conditions of deposition.

Redox-sensitive trace elements behave distinctly under different redox conditions, and are thus widely utilized as redox indicators in modern and ancient environments[36-38]. Enrichment patterns of manganese, rhenium, uranium and molybdenum are briefly introduced here. Mn forms highly insoluble Mn(III) or Mn(IV) (hydr)oxide minerals in oxic environments, which are rapidly deposited in particulate form[36,39]. By contrast, under dysoxic-anoxic conditions Mn is reduced to dissolved Mn(II) phases, and released from sediments[38]. Unlike Mn, Re is primarily present as soluble Re(VII) species in oxygenated settings, and is reduced to insoluble Re(IV) minerals under dysoxic conditions and sequestered in the sediments[40,41]. Rhenium starts to accumulate in dysoxic sediments where the oxygen penetrate depth is shallow (~1 cm), and thus may be used to track the development of dysoxic conditions at the sediment-water interface[41,42]. Uranium is mainly present as soluble U(VI) species under oxygenated water column conditions, but is reduced to insoluble U(IV) species under anoxic conditions and sequestered in anoxic sediments[43,44]. Molybdenum occurs as dissolved Mo(VI) under oxygenated water column conditions, and can be absorbed to Mn-Fe oxides, potentially leading to relatively low enrichments under ferruginous conditions[45,46]. However, under euxinic conditions, molybdate is reduced to particle reactive thiomolybdate species, which can result in strong enrichments in sulfidic sediments[41,47].

These combined systematics allow a detailed, integrated assessment of the bottom water redox conditions during deposition of the studied sections. For unit 4, consideration of the relationships between Fe_{HR}/Fe_T ratios and U_{EF} values (see main text) provides nuanced insight into the redox characteristics of the depositional setting, with the combined data being entirely consistent with deposition periodically within and below an oxygen minimum zone[48]. For unit 1, further robust support for the validity of our Fe speciation data is obtained by considering relationships with redox sensitive

trace metals through this unit, using the more extensive data-set of Wang et al.[4] (Fig. S4). This approach clearly demonstrates that as Fe_{HR}/Fe_{T} ratios increase above the anoxic threshold, U_{EF} values, as an independent indicator of bottom water anoxia[43,44], also increase to values that are substantially higher than the upper continental crust value of 1 (Fig. S4A), providing strong support for dominantly anoxic depositional conditions. Next, we consider Mo_{EF} values, which provide an independent indicator of water column euxinia[47,49,50]. The majority of samples plot in the ‘possibly euxinic’ and ‘euxinic’ fields on an Fe speciation phase-space crossplot, suggesting dominantly euxinic depositional conditions, which is particularly the case for samples with Mo_{EF} values >2 (Fig. 2B). There is also a progressive increase in average Fe_{py}/Fe_{HR} ratios when the Mo_{EF} data are grouped, which is consistent with variability in the intensity of euxinia, whereby more intense euxinia promotes higher Fe_{py}/Fe_{HR} ratios and increasingly elevated Mo_{EF} values. Taken together, the close degree of consistency between Fe speciation and redox sensitive trace metal systematics strongly supports robust geochemical signals, which are dominantly controlled by water column redox dynamics.

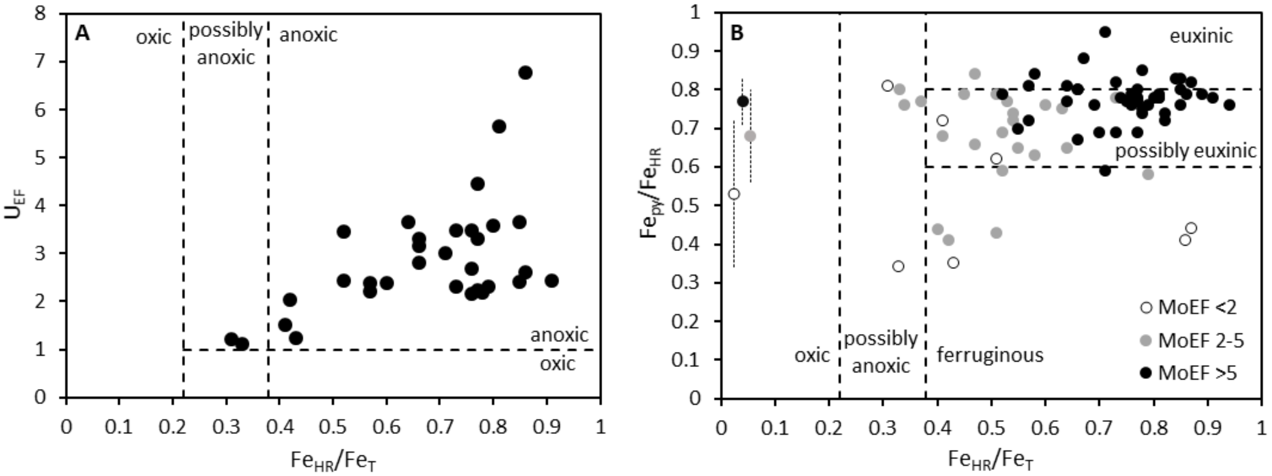

Fig. S4. Paleoredox proxy crossplots for unit 1. A. U_{EF} values as a function of Fe_{HR}/Fe_{T}; B. Fe_{py}/Fe_{HR} as a function of Fe_{HR}/Fe_{T}, with data grouped based on molybdenum enrichment factors (Mo_{EF}). Average values (± 1sd) for each Mo_{EF} group are displayed in the ‘oxic’ field. All data are from Wang et al.[4].

In addition, in Fig. S5 we show that the periodic intervals of relative Fe_{HR} depletion in Section B are not due to transfer of Fe_{HR} to Fe-rich clay minerals during diagenesis[24], since the close agreement between trends in Fe_{HR}/Fe_T and Fe_T/Al unequivocally demonstrate that the observed cyclicity is a primary feature, with both redox parameters independently documenting enhanced Fe deposition in the red mudstone intervals.

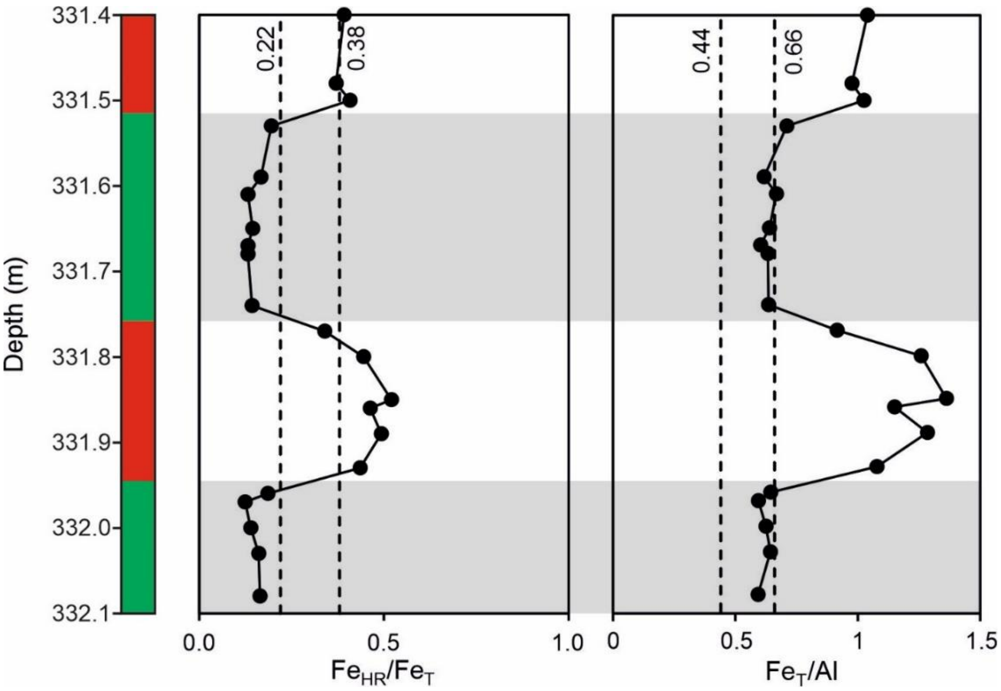

Fig. S5. Geochemical profiles for Fe_{HR}/Fe_T and Fe_T/Al through Section B. Dashed lines on Fe_{HR}/Fe_T plot represent the traditional boundaries for identifying oxic (<0.22) and anoxic (>0.38) depositional conditions in normal marine (i.e., not above and below an oxygen minimum zone (OMZ) as in the present case) settings[24]. Dashed lines on Fe_T/Al plot represent the normal range (0.44–0.66) for oxic depositional conditions[26]. Shading corresponds to lithologic changes between red and green mudstones, in response to redox variations.

Paleoclimate proxies

Potassium is a highly mobile element, and its cycling is predominantly controlled by chemical weathering, sedimentation and hydrothermal circulation. The input of K to the ocean is controlled by the

relative intensity of continental weathering and the hydrothermal flux, while the removal of K is closely related to chemical exchange involving clays in sediments and low temperature basalt alteration[51,52]. In the weathering profile, K is typically enriched in illite, but is preferentially lost from sediments during chemical weathering due to its high mobility[53,54]. By contrast, Al is a refractory element commonly associated with clay minerals, and therefore K/Al ratios are sensitive to chemical weathering intensity, with relatively lower values generally indicating higher intensity of chemical weathering[55,56].

The magnitude of K/Al ratios will also vary in terms of the primary ratio in the source material supplied to the region, while reverse weathering in marine sediments can also promote K drawdown from seawater[57]. However, it is difficult to envisage why reverse weathering would give the dynamic changes in K/Al ratios observed in Section A, particularly given the close correspondence to changes in multiple independent geochemical proxies (Fig 2). Hence, high K/Al ratios in the source region provides the most likely explanation for the elevated ratios relative to UCC (Fig. 2). Nevertheless, we stress here that our focus is on relative changes in K/Al ratios (as an indication of relative changes in chemical weathering), rather than absolute values.

Similar to Al, Ti is relatively resistant to chemical weathering and is commonly concentrated in heavy minerals, generally in association with coarser-grained sediments[58,59]. As a result, Ti/Al ratios are commonly impacted by the intensity of transport processes, either in air or by rivers[53,59]. However, detailed studies have shown that aeolian transport is a significant pathway for delivery of Ti to marine sediments, with Ti/Al ratios indicating relative wind strength in arid regions[54,56,60].

Phosphorus cycling

Phosphorus bioavailability in the water column is dominantly controlled by a balance between continental weathering inputs, and outputs to sediments in association with organic matter, iron (oxyhydr)oxides and as authigenic phases (e.g., carbonate fluorapatite and vivianite), with detrital apatite

representing a non-bioavailable form of P that is transported to the marine environment and buried essentially unaltered[61,62]. In addition, P burial in marine sediments is highly redox dependent[63,64]. Under oxic conditions, aerobic decomposition of organic matter releases organic P (P_{org}) from sediments, which is then commonly trapped in sediments via 'sink-switching' to carbonate fluorapatite (P_{aut}) or via adsorption to iron (oxyhydr)oxides (P_{Fe})[65,66]. Similarly, iron minerals in ferruginous sediments can potentially act as an effective 'iron trap' for phosphorus[67,68]. However, P fixation is largely diminished in anoxic sulfidic sediments, where P is preferentially regenerated from organic matter during anaerobic degradation and the reduction of Fe (oxyhydr)oxide minerals diminishes the iron trap for P, potentially resulting in elevated P recycling back to water column[64,69,70]. However, as with oxic and ferruginous sediments, the recycled flux of P may be moderated by the formation of authigenic carbonate fluorapatite[62,71]. In addition, reduced iron phosphate minerals (e.g., vivianite) may form in low sulfate euxinic settings due to insufficient sulfide availability in pore waters to buffer dissolved Fe(II) via the formation of pyrite, and this has been suggested to be a potential major P sink in the low sulfate Mesoproterozoic ocean[72].

Determination of P phase partitioning and quantifying the recycled flux of P_{reac} in Section A

The phase partitioning of P was determined via a modified SEDEX method, specifically tailored for ancient rocks[73,74]. Operational details are provided in Table S1, which includes Relative Standard Deviations (RSDs) for each step.

The phase partitioning approach allows quantification of P_{org} and P_{reac}, whereby P_{Fe1}, P_{Fe2} and P_{mag} are summed to give P_{Fe}, and P_{reac} is calculated as $P_{Fe} + P_{auth} + P_{org}$. As shown in Fig. 2A, relatively lower TOC/P_{reac} ratios are observed in highly euxinic intervals of Section A, which potentially indicates a lower degree of P recycling back to the water column. However, the absolute flux of recycled P is also related to the amount of organic matter and associated organic P that was initially deposited. Therefore,

we calculate a minimum estimate for the amount of recycled P, by considering the measured concentration of TOC in the sediment (which will be substantially lower than the original depositional concentration of TOC, hence giving a minimum estimate of recycled P), and assuming this was buried with an original TOC/P_{org} ratio equivalent to the Redfield ratio (106/1). This gives an estimate of the total initial concentration of P_{org} in the sediment. Making the reasonable assumption that under euxinic conditions, the majority of P_{reac} was derived from P_{org}, this equates to a minimum initial concentration of P_{reac} in the sediment. Subtraction of the measured concentration of P_{reac} then gives a minimum amount of P_{reac} recycled back to the water column (Fig. S6). This exercise shows that despite a higher relative degree of recycling under more weakly euxinic conditions (Fig. 2A), the actual amount of P that was recycled back to the water column was approximately constant across the weakly and highly euxinic intervals. By extension, since there is no evidence for a significant change in sedimentation rate between the highly and weakly euxinic intervals, this suggests that rates of P recycling were also approximately constant, and indeed, relatively minor changes in sedimentation rate are unlikely to alter this conclusion.

Table S1. Sequential P extraction steps. RSDs are based on replicate extractions.

Step	Extractant	Target P phase	RSD (%)
I	10 ml sodium citrate/sodium bicarbonate/ sodium dithionite solution (8 h)	P_{Fe1}: Fe(oxyhydr)oxide-bound P	5
II	10 ml 1 M acetate sodium (pH 4, 6 h)	P_{auth}: carbonate-associated P, authigenic apatite and biogenic apatite	2
III	10 ml 1 M HCl (16 h)	P_{det}: detrital apatite and other inorganic P phases	1
IV	10 ml ammonium oxalate (6 h)	P_{mag}: magnetite-bound P	2
V	10 ml sodium citrate/sodium dithionite/acetic acid (6 h)	P_{Fe2}: hematite-bound P	18
VI	Ash at 550ºC<br>10 ml 1 M HCl (16 h)	P_{org}: organic P	1

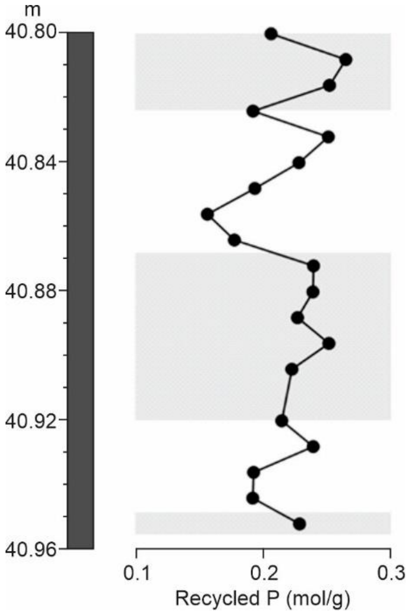

Fig. S6. Estimated minimum concentration of P recycled back to the water column in Section A. Highly euxinic intervals are indicated by grey shading.

Depositional Models

In Section A, the combination of Fe speciation and Mo-U enrichment systematics suggest a euxinic depositional environment. Periodic fluctuations in Mo_{EF} and pyrite $\delta^{34}S$ data further indicate alternations between weakly and highly euxinic conditions, and we expand upon this interpretation here. In Section A, intervals of more intense chemical weathering (as indicated by lower K/Al ratios) occur alongside higher Mo_{EF} values (Fig. 2). Higher intensity chemical weathering increases the oceanic influx of both sulfate and Fe_{HR} (in the form of Fe (oxyhydr)oxides) from pyrite weathering on land, but Fe_{HR} is proportionately increased relative to sulfate as weathering intensity increases, due to additional release of Fe_{HR} from parent silicate minerals[75-78]. This increasingly focuses the locus of Fe_{HR} sulfidation (i.e., pyrite formation) to deeper in the sediment pile, with proportionately less sulfidation occurring in the water column[77,78]. Under constant sulfide concentrations in the water column, this would result in increased pyrite $\delta^{34}S$ values as proportionately more of the pyrite would be formed under closed system

conditions in the sediment[79]. However, our data show decreased pyrite $\delta^{34}S$ values in the intervals of more intense chemical weathering (Fig. 2). Along with the higher Mo_{EF} values, this suggests that sulfide concentrations were higher in the water column, since rates of Fe_{HR} sulfidation are directly related to sulfide concentrations[80,81], and enhanced sulfidation in the water column is required to explain the lower pyrite $\delta^{34}S$ values.

Elevated P input from runoff and regeneration from anoxic sediments under euxinic conditions promoted enhanced primary productivity, thus facilitating a eutrophic depositional setting. During intervals when weathering intensity was decreased, weakly euxinic conditions developed, ultimately driving less organic carbon burial. These cycles occurred over regular intervals, but some of the geochemical proxies show a slight offset. For example, at the base of the upper weakly euxinic interval (main text Fig. 2A), TOC concentrations start to drop before Mo_{EF} values drop, reflecting a delayed response in terms of water column sulfide concentrations decreasing in the water column, which might be expected as the standing sulfide reservoir would first need to be depleted.

For Section B, opposing trends in Fe_{HR}/Fe_T and U_{EF} occur (Fig. 2B). As observed in the modern Peru margin[82], reducing sediments within the OMZ can act as an iron shuttle, where Fe is reductively remobilized then re-precipitated in better oxygenated waters below the OMZ. We propose a similar depositional scenario for unit 4 in the Xiamaling Formation. Green mudstones with lower Fe_{HR} concentrations, but dominantly as reduced phases, were deposited within the OMZ, which is supported by relatively enriched U. Red mudstones were most likely deposited beneath the OMZ, following re-precipitation of mobilized Fe^{2+} as Fe (oxyhydr)oxides. In these red mudstone intervals, enrichments in Re and depletion in Mn suggest that the bottom water was most likely oxygen-depleted, rather than fully oxic.

Biogeochemical model

Full equations for the biogeochemical model are shown below, and model species and fluxes are presented in Tables S2-S3. This is a modification of the model from Dal Corso et al.[83] and this section repeats many of the definitions from that work and other texts. For completeness, this section also repeats some parts of the methods section from the main text.

Table S2. Model species.

Description	Name	Exists in	Size at present
Surface ocean water shelf 1	W_{s1}	Surface Ocean	1.5×10^{16} m^3
Surface ocean water shelf 2	W_{s2}	Surface Ocean	1.5×10^{16} m^3
High-latitude water	W_h	High Latitude	1.35×10^{16} m^3
Deep water	W_d	Deep ocean	1.35×10^{18} m^3
Atmospheric CO_2	CO_{2_a}	Atmosphere	5×10^{16} mol C
Surface ocean 1 DIC	DIC_{s1}	Surface Ocean	3×10^{16} mol C *
Surface ocean 2 DIC	DIC_{s2}	Surface Ocean	3×10^{16} mol C *
High-latitude DIC	DIC_h	High Latitude	3×10^{16} mol C *
Deep ocean DIC	DIC_d	Deep ocean	3×10^{18} mol C *
Surface ocean alkalinity 1	ALK_{s1}	Surface Ocean	3×10^{16} mol $CaCO_3$ equiv. *
Surface ocean alkalinity 2	ALK_{s2}	Surface Ocean	3×10^{16} mol $CaCO_3$ equiv. *
High-latitude alkalinity	ALK_h	High Latitude	3×10^{16} mol $CaCO_3$ equiv. *
Deep ocean alkalinity	ALK_d	Deep ocean	3×10^{18} mol $CaCO_3$ equiv. *
Surface ocean phosphate	P_s	Surface Ocean	3×10^{13} mol P
High-latitude phosphate	P_h	High Latitude	3×10^{13} mol P
Deep ocean phosphate	P_d	Deep ocean	3×10^{15} mol P

*Starting values chosen close to equilibrium values, model equilibrates to DIC ≈ 2 mM and ALK ≈ 2.2 mM, roughly approximate to the modern ocean. Other values follow Sarmiento and Toggweiler[84] and Lenton et al.[85].

Model fluxes

Model fluxes, with equations and present values are shown in Table S3. Transfer fluxes follow a simple concentration relationship, air sea exchange follows Walker and Kasting[86], carbonate burial (net accumulation) follows Rampino and Caldeira[87], and all other fluxes are chosen from recent carbon cycle models[88].

Table S3. Model fluxes.

Description	Name	Equation	Size at present
Transfer fluxes	$tran_{ij}$	$C_i f_{circ} U_{frac}$	Multiple
Air sea exchanges	f_{airsea_j}	$A_j M_{atm}(\frac{pCO_{2_a} - pCO_{2_j}}{\tau_{oa}})$	Multiple
Silicate weathering	f_{silw}	$W_i \cdot Wfrac_i \cdot \left(k_{basw} f_{T_{bas}} + k_{granw} f_{T_{gran}}\right)$	8×10^{12} mol C yr^{-1}
Carbonate weathering	f_{carbw}	$W_i \cdot Wfrac_i \cdot k_{carbw} f_{Tcarb}$	8×10^{12} mol C yr^{-1}
Oxidative weathering	f_{oxidw}	$W_i \cdot Wfrac_i \cdot k_{oxidw}(RO_2)^{0.5}$	7.75×10^{12} mol C yr^{-1}
Phosphate weathering	f_{phosw}	$W_i \cdot Wfrac_i \cdot k_{phosw}$	4.17×10^{10} mol P yr^{-1}
Carbonate degassing	f_{ccdeg}	k_{ccdeg}	8×10^{12} mol C yr^{-1}
Organic C degassing	f_{ocdeg}	k_{ocdeg}	1.25×10^{12} mol C yr^{-1}
Marine carbonate burial	f_{mccb}	$k_{mccb} \frac{(\Omega - 1)^{1.7}}{\Omega_0}$	16×10^{12} mol C yr^{-1}
Marine organic C burial	f_{mocb}	$C_{sed_i} \cdot BE$	4.5×10^{12} mol C yr^{-1}
Land organic C burial	f_{locb}	k_{locb}	4.5×10^{12} mol C yr^{-1}
Evaporite dissolution	$f_{evapdis}$	$k_{evapdis}$	Varied in experiments
Evaporite deposition	$f_{evapdep}$	$k_{evapdep}$	Varied in experiments

Non-flux calculations

Atmospheric CO2 volume ratio:

$$O_2ppm = 280\frac{CO_{2_a}}{CO_{2_{a_0}}} \tag{S1}$$

where CO_{2_a} is atmospheric CO_2 in moles, and $CO_{2_{a_0}}$ is this value at present day.

Global average surface temperature:

$$GAST = 288 + k_{clim}\left(\frac{\log\left(\frac{CO_2ppm}{280}\right)}{\log(2)}\right) \tag{S2}$$

where t_{clim} is climate sensitivity to doubling CO_2. Low-latitude surface temperature (T_s) is assumed to scale by $\frac{2}{3}$ times global temperature change, and both high-latitude (T_h) and deep ocean (T_d) temperature are assumed to follow global temperature change.

Carbonate speciation:

Effective equilibrium constants are calculated following previous studies[86,88]. These consider only temperature dependencies, omitting those on pressure and salinity.

$$K_{carb} = 5.75 \times 10^{-4} + 6 \times 10^{-6}(T_j - 278) \tag{S3}$$

$$K_{CO_2} = 0.035 + 0.0019(T_j - 278)\ \text{PAL m}^3\ \text{mol}^{-1} \tag{S4}$$

Dissolved carbon species are then calculated following[48]:

$$[HCO_3^-]_j = DIC_j - \frac{\sqrt{DIC_j^2 - ALK_j(2DIC_j - ALK_j)(1-4K_{carb})}}{1-4K_{carb}} \tag{S5}$$

$$[CO_3^{2-}]_j = \frac{ALK_j - [HCO_3^-]_j}{2} \tag{S6}$$

$$pCO_{2_j} = \frac{K_{CO_2}[HCO_3^-]^2}{[CO_3^{2-}]} \tag{S7}$$

Calcium carbonate saturation state:

$$\Omega_j = \frac{[Ca]_j[CO_3^{2-}]_j}{K_{sp}} \tag{S8}$$

where Ω_j is the $CaCO_3$ saturation state in box j and K_{sp} is the solubility product. [Ca] and $[CO_3^{2-}]$ are concentrations.

Terrestrial chemical weathering

Temperature dependence of basalt and granite weathering:

$$f_{T_{bas}} = e^{0.0608(GAST-288)\left(1+0.038(GAST-288)\right)^{0.65}} \quad \text{(S9)}$$

$$f_{T_{gran}} = e^{0.0724(GAST-288)\left(1+0.038(GAST-288)\right)^{0.65}} \quad \text{(S10)}$$

Temperature dependence of carbonate weathering:

$$f_{T_{carb}} = 1 + 0.087(GAST - 288) \quad \text{(S11)}$$

Weathering constants:

$$k_{basw} = 2.4 \times 10^{12} \text{ mol yr}^{-1} \quad \text{(S12)}$$

$$k_{granw} = 5.6 \times 10^{12} \text{ mol yr}^{-1} \quad \text{(S13)}$$

Weathering cyclicity:

To replicate the orbital forcing, a sinusoidal multiplier is applied to weathering fluxes into Shelf 1 and Shelf 2. These are set to oscillate on approximately 15 kyr and 60 kyr timeframes, where *t* is time in model years:

$$W_{15} = 1 + \sin(4 \cdot t \cdot 10^{-4}) \quad \text{(S14)}$$

$$W_{60} = 1 + \sin(t \cdot 10^{-4}) \quad \text{(S15)}$$

Weathering fluxes for silicates, carbonate, phosphate and organic matter are defined as a product of the weathering constant, cyclicity, temperature dependence and local weathering fraction *Wfrac*.

Remineralization and organic carbon burial

A simplified remineralization scheme is applied to the model sediment-water interface to demonstrate how altered carbon supply in each shelf could drive cyclicity in the severity of euxinic

conditions or in the extent of a ferruginous OMZ. We first calculate the available organic carbon from the overall model C_{org} burial flux at the present day (k_{mocb}), a relationship to local shelf P concentration relative to the present day, and a simple burial efficiency (*BE*) set to 10%.

$$C_{sed_i} = \frac{k_{mocb}}{2} \cdot \frac{P_{Si}}{P_{S_0}} \cdot \frac{1}{BE} \tag{S16}$$

For our test case we assume a constant fraction of the available carbon is processed through Fe(III) reduction and the remainder is processed via SO_4 reduction:

$$remin_{Fe_i} = 0.5 \cdot C_{sed_i} \cdot (1 - BE) \tag{S17}$$

$$remin_{S_i} = C_{sed_i} \cdot (1 - BE) \cdot \left(1 - remin_{Fe_i}\right) \tag{S18}$$

Organic carbon burial is calculated as:

$$mocb_{sed_i} = C_{sed_i} \cdot BE \tag{S19}$$

Fixed parameters are shown in Table S4.

Table S4. Fixed parameters.

Description	Name	Size at present
Thermohaline speed	f_{circ}	20 Sv
Relative area of low-latitude surface ocean	A_s	0.85
Relative area of high-latitude surface ocean	A_h	0.15
Present day moles of atmospheric CO_2	M_{atm}	5×10^{16} mol C
Timescale parameter for gas exchange	τ_{oa}	10 years
Long-term climate sensitivity	k_{clim}	5 K
Calcium carbonate solubility product	K_{sp}	0.8 $mmol^2\ kg^{-2}$ *
Present day $CaCO_3$ saturation state	Ω_0	3

*chosen within ocean range (0.43–1.15)[89] to achieve reasonable DIC and ALK at present. Other parameters follow[85,87,90]. Long-term climate sensitivity[91] is larger than equilibrium climate sensitivity (ECS), and appears to be around 5K during the Phanerozoic[92].

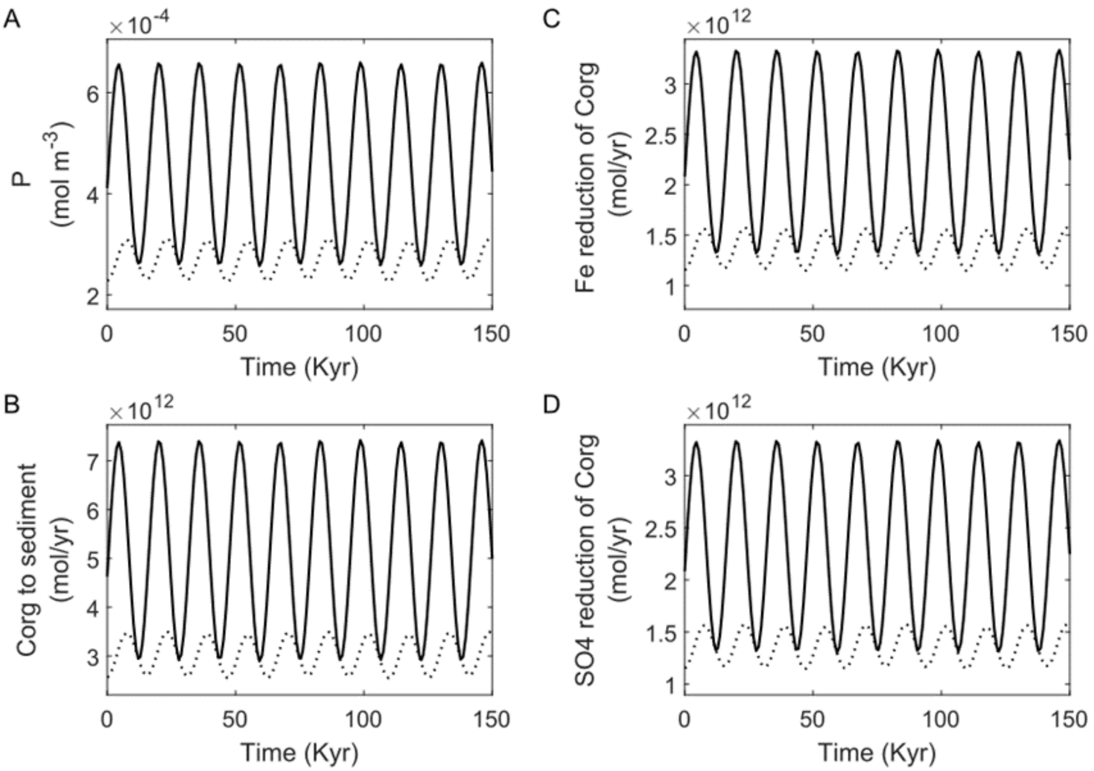

Fig. S7. Alternative model results for equal upwelling between shelf/surface ocean boxes. Here, 50% of total upwelling is routed into each of the surface ocean boxes so that differences between the shelves are driven by weathering alone. Otherwise, the model setup is identical to that shown in Fig. 4. (**A**) A high weathering flux on Shelf 1 is set to oscillate at periods of 15 kyr (solid line), while a low weathering flux oscillates synchronously on Shelf 2 (dashed line). (**B**) High and variable organic carbon burial rates (C_{org}) occur on Shelf 1, while low but variable rates occur on Shelf 2. (**C**) High and variable C_{org} consumption rates occur due to Fe(III) reduction on Shelf 1, with very low but variable remineralization rates by Fe(III) reduction on Shelf 2. (**D**) High and variable C_{org} consumption rates occur due to sulfate reduction on Shelf 1, with very low but variable remineralization rates by sulfate reduction on Shelf 2.

Differential equations

The following equations track the 12 non-water species from Table S2.

Atmospheric CO_2:

$$\frac{d(CO_{2_a})}{dt} = -f_{airsea_{s1}} - f_{airsea_{s2}} - f_{airsea_h} + f_{ccdeg} + f_{ocdeg} + f_{oxidw} - f_{locb} - f_{carbw} - 2f_{silw} \quad (S20)$$

Low-latitude surface ocean DIC Shelf 1

$$\frac{d(DIC_{s1})}{dt} = f_{airsea_{s1}} + tran_{DIC_{ds1}} - tran_{DIC_{sh1}} + 2f_{carbw1} + 2f_{silw1} - f_{mccb1} - f_{mocb1} \quad (S21)$$

Low-latitude surface ocean DIC Shelf 2

$$\frac{d(DIC_{s2})}{dt} = f_{airsea_{s2}} + tran_{DIC_{ds2}} - tran_{DIC_{sh2}} + 2f_{carbw2} + 2f_{silw2} - f_{mccb2} - f_{mocb2} \quad (S22)$$

High-latitude surface ocean DIC

$$\frac{d(DIC_h)}{dt} = f_{airsea_{h1}} + tran_{DIC_{s1h}} + tran_{DIC_{s2h}} - tran_{DIC_{hd}} \quad (S23)$$

Deep ocean DIC

$$\frac{d(DIC_d)}{dt} = tran_{DIC_{hd}} - tran_{DIC_{ds1}} - tran_{DIC_{ds2}} \quad (S24)$$

Low-latitude surface ocean alkalinity Shelf 1

$$\frac{d(ALK_{s1})}{dt} = tran_{ALK_{ds1}} - tran_{ALK_{s1h}} + 2f_{carbw1} + 2f_{silw1} - 2f_{mccb1} \quad (S25)$$

Low-latitude surface ocean alkalinity Shelf 2

$$\frac{d(ALK_{s2})}{dt} = tran_{ALK_{ds2}} - tran_{ALK_{s2h}} + 2f_{carbw2} + 2f_{silw2} - 2f_{mccb2} \quad (S26)$$

High-latitude surface ocean alkalinity

$$\frac{d(ALK_h)}{dt} = tran_{ALK_{s1h}} + tran_{ALK_{s2h}} - tran_{ALK_{hd}} \quad (S27)$$

Deep ocean alkalinity

$$\frac{d(ALK_d)}{dt} = tran_{ALK_{hd}} - tran_{ALK_{ds1}} - tran_{ALK_{ds2}} \quad (S28)$$

Low-latitude surface ocean P Shelf 1

$$\frac{d(P_{s1})}{dt} = tran_{P_{ds1}} - tran_{P_{s1h}} + f_{phosw1} - f_{phosb1} \quad (S29)$$

Low-latitude surface ocean P Shelf 2

$$\frac{d(P_{s2})}{dt} = tran_{P_{ds2}} - tran_{P_{s2h}} + f_{phosw2} - f_{phosb2} \quad \text{(S30)}$$

High-latitude surface ocean P

$$\frac{d(P_h)}{dt} = tran_{P_{s1h}} + tran_{P_{s2h}} - tran_{P_{hd}} \quad \text{(S31)}$$

Deep ocean P

$$\frac{d(P_d)}{dt} = tran_{P_{hd}} - tran_{P_{d1}} - tran_{P_{ds2}} \quad \text{(S32)}$$

Model solution

The model is solved in MATLAB using the ODE15s variable-order method for stiff systems. Code is freely available at github.com/bjwmills.

Supplementary references

1. Meng, Q., Wei, H., Qu, Y. & Ma, S. Stratigraphic and sedimentary records of the rift to drift evolution of the northern North China craton at the Paleo-to Mesoproterozoic transition. *Gondwana Res.* **20**, 205-218 (2011).
2. Qu, et al. Geological characteristics and tectonic significance of unconformities in Mesoproterozoic successions in the northern margin of the North China Block. *Geosci. Front.* **5**, 127-138 (2014).
3. Zhang, S. et al. Orbital forcing of climate 1.4 billion years ago. *Proc. Natl Acad. Sci. USA* **112**, 1406-1413 (2015).
4. Wang, X. et al. Oxygen, climate and the chemical evolution of a 1400 million year old tropical marine setting. *Am. J. Sci.* **317**, 861-900 (2017).
5. Mutti, E. Distinctive thin-bedded turbidite facies and related depositional environments in the Eocene Hecho group (South-central Pyrenees, Spain). *Sedimentology* **24**, 107-131 (1977).
6. Tinterri, R. Combined flow sedimentary structures and the genetic link between sigmoidal- and hummocky cross stratification. *GeoActa* **10**, 1-43 (2011).
7. Privat, A. M-L., Hodgson, D. M., Jackson, C. A-L., Schwarz, E. & Peakall, J. Evolution from syn-rift carbonates to early post-rift deep-marine intraslope lobes: the role of rift basin physiography on sedimentation patterns. *Sedimentology* **68**, 2563-2605 (2021).

8. Clare, M. A., Talling, P. J., Challenor, P., Malgesini, G. & Hunt, J. Distal turbidites reveal a common distribution for large (> 0.1 km3) submarine landslide recurrence. *Geology* **42**, 263-266 (2014).
9. Clare, M. A., Talling, P. J. & Hunt, J. E. Implications of reduced turbidity current and landslide activity for the initial Eocene Thermal Maximum – evidence from two distal, deep-water sites. *Earth Planet. Sci. Lett.* **420**, 102-115 (2015).
10. Li, M., Hinnov, L. & Kump, L. Acycle: Time-series analysis software for paleoclimate research and education. *Comput and Geosci.* **127**, 12-22 (2019).
11. Zhang, S. et al. in *Ancient Supercontinents and the Paleogeography of Earth*, Pesonen, L. J., Salminen, J., Elming, S. Å., Evans, D. A. & Veikkolainen, T. Eds. (Elsevier, 2021), pp. 333-376.
12. Zhang, S. et al. Pre-Rodinia supercontinent Nuna shaping up: A global synthesis with new paleomagnetic results from North China. *Earth Planet. Sci. Lett.* **353**, 145-155 (2012).
13. Chen, L., Huang, B., Yi, Z., Zhao, J. & Yan, Y. Paleomagnetism of ca. 1.35 Ga sills in northern North China Craton and implications for paleogeographic reconstruction of the Mesoproterozoic supercontinent. *Precambrian Res.* **228**, 36-47 (2013).
14. Evans, D. A. & Mitchell, R. N. Assembly and breakup of the core of Paleoproterozoic–Mesoproterozoic supercontinent Nuna. *Geology* **39**, 443-446 (2011).
15. Pisarevsky, S. A., Elming, S.Å., Pesonen, L. J. & Li, Z. Mesoproterozoic paleogeography: Supercontinent and beyond. *Precambrian Res.* **244**,207-225 (2014).
16. Elming, S. Å., Salminen, J. & Pesonen, L. J. in *Ancient Supercontinents and the Paleogeography of Earth*. Pesonen, L. J., Salminen, J., Elming, S. Å., Evans, D. A. & Veikkolainen, T. Eds. (Elsevier 2021), pp. 499-548.
17. Elston, D. P., Enkin, R. J., Baker, J. & Kisilevsky, D. K. Tightening the Belt: Paleomagnetic-stratigraphic constraints on deposition, correlation, and deformation of the Middle Proterozoic (ca. 1.4 Ga) Belt-Purcell Supergroup, United States and Canada. *Geol. Soc. Am. Bull.* **114**, 619-638 (2002).
18. Broccoli, A. J., Dahl, K. A. & Stouffer. R. J. Response of the ITCZ to Northern Hemisphere cooling. *Geophys. Res. Lett.* **33**, (2006).
19. Waliser, D. E. & Gautier, C. A satellite-derived climatology of the ITCZ. *J. Clim.* **6**, 2162-2174 (1993).

20. Schneider, T., Bischoff, T. & Haug, G. H. Migrations and dynamics of the intertropical convergence zone. *Nature* **513**, 45-53 (2014).

21. Haug, G. H., Hughen, K. A., Sigman, D. M., Peterson, L. C. & Röhl, U. Southward migration of the intertropical convergence zone through the Holocene. *Science* **293**, 1304–1308 (2001).

22. Koutavas, A. & Lynch-Stieglitz, J. in *The Hadley Circulation: Present, Past, and Future*. Diaz, H. F. & Bradley, R. S. Eds. (Kluwer Academic, 2004), pp. 347–369.

23. Poulton, S. W. & Canfield, D. E. Ferruginous conditions: a dominant feature of the ocean through Earth's history. Elements 7, 107-112 (2011).

24. Poulton, S. W. The iron speciation paleoredox proxy. (Cambridge University Press, 2021).

25. Pasquier, V., Fike, D. A., Révillon, S. & Halevy, I. A global reassessment of the controls on iron speciation in modern sediments and sedimentary rocks: A dominant role for diagenesis. *Geochim. Cosmochim. Acta* **335**, 211-230 (2022).

26. Clarkson, M. O., Poulton, S. W., Guilbaud, R. & Wood, R. A. Assessing the utility of Fe/Al and Fe-speciation to record water column redox conditions in carbonate-rich sediments. *Chem. Geol.* **382**, 111-122 (2014).

27. Canfield, D. E., Lyons, T. W. & Raiswell, R. A model for iron deposition to euxinic Black Sea sediments. *Am. J. Sci. **296***, 818-834 (1996).

28. Raiswell et al. The iron paleoredox proxies: A guide to the pitfalls, problems and proper practice. *Am. J. Sci.* **318**, 491-526 (2018).

29. Wei, G. et al. A chemical weathering control on the delivery of particulate iron to the continental shelf. *Geochim. Cosmochim. Acta* **308**, 204-216 (2021).

30. Poulton, S. W. & Raiswell, R. The low-temperature geochemical cycle of iron: from continental fluxes to marine sediment deposition. *Am. J. Sci.* **302**, 774-805 (2002).

31. Raiswell, R., Newton, R. & Wignall, P. B. An indicator of water-column anoxia: resolution of biofacies variations in the Kimmeridge Clay (Upper Jurassic, UK). *J. Sediment. Res.* **71**, 286-294.

32. Raiswell, R.Turbidite depositional influences on the diagenesis of Beecher’s trilobite bed and the Hunsrück slate; Sites of soft tissue pyritization. *Am. J. Sci.* **308**, 105-129 (2008).

33. He. T., Wignall, P. B., Newton, R. J., Atkinson, J. W., Keeling, J. F. J., Xiong, Y. & Poulton, S. W. Extensive marine anoxia in the European epicontinental sea during the end-Triassic mass extinction. *Glob. Planet. Change* **210**, 103771 (2022).

34. Kang, J., Gill, B., Reid. R., Zhang. F. & Xiao, S. Nitrate limitation in early Neoproterozoic oceans delayed the ecological rise of eukaryotes. *Sci. Adv.* **9**, eade9647 (2023).

35. Li, S., Wignall, P. B., Xiong, Y. & Poulton, S. W. Calibration of redox thresholds in black shale: Insight from a stratified Mississippian basin with warm saline bottom waters. *Geol. Soc. Am. Bull.* in press.

36. Calvert, S. E. & Pedersen, T. F. Geochemistry of recent oxic and anoxic marine sediments: implications for the geological record. *Mar. Geol.* **113**, 67-88 (1993).

37. Morford, J. L. & Emerson, S. The geochemistry of redox sensitive trace metals in sediments. *Geochim. Cosmochim. Acta*, **63**, 1735-1750 (1999).

38. Algeo, T. J. & Maynard, J. B. Trace-element behavior and redox facies in core shales of Upper Pennsylvanian Kansas-type cyclothems. *Chem. Geol*. **206**, 289-318 (2004).

39. Sholkovitz, E. R., Shaw, T. J. & Schneider, D. L. The geochemistry of rare earth elements in the seasonally anoxic water column and porewaters of chesapeake bay. *Geochim. Cosmochim. Acta* **56**, 3389-3402 (1992).

40. Colodner, D. et al. The geochemical cycle of rhenium: a reconnaissance. *Earth Planet. Sci. Lett.* **117**, 205-221 (1993).

41. Crusius, J., Calvert, S., Pedersen, T. & Sage, D. Rhenium and molybdenum enrichments in sediments as indicators of oxic, suboxic and sulfidic conditions of deposition. *Earth Planet. Sci. Lett.* **145**, 65-78 (1996).

42. Morford, J. L., Emerson, S. R., Breckel, E. J. & Kim, S. H. Diagenesis of oxyanions (V, U, Re, and Mo) in pore waters and sediments from a continental margin. *Geochim. Cosmochim. Acta* **69**, 5021-5032 (2005).

43. Anderson, R. F., Fleisher, M. Q. & LeHuray, A. P. Concentration, oxidation state, and particulate flux of uranium in the Black Sea. *Geochim. Cosmochim. Acta* **53**, 2215-2224 (1989).

44. Klinkhammer, G. & Palmer, M. Uranium in the oceans: where it goes and why. *Geochim. Cosmochim. Acta* **55**, 1799-1806 (1991).

45. Algeo, T. J. & Tribovillard, N. Environmental analysis of paleoceanographic systems based on molybdenum–uranium covariation. *Chem. Geol.* **268**, 211-225 (2009).

46. Tribovillard, N., Algeo, T.J., Baudin, F. & Riboulleau, A. Analysis of marine environmental conditions based on molybdenum–uranium covariation—Applications to Mesozoic paleoceanography. *Chem. Geol.* **324**, 46-58 (2012).

47. Erickson, B. E. & Helz, G. R. Molybdenum (VI) speciation in sulfidic waters: stability and lability of thiomolybdates. *Geochim. Cosmochim. Acta* **64**, 1149-1158 (2000).
48. Scholtz, F., Hensen, C., Noffke, A., Rohde, A., Liebetrau, V. & Wallmann, K. Early diagenesis of redox sensitive trace metals in the Peru upwelling area – response of ENSO-related oxygen fluctuations in the water column. *Geochim. Cosmochim. Acta* **75**, 7257-7276 (2011).
49. Helz, G. R. et al. Mechanism of molybdenum removal from the sea and its concentration in black shales: EXAFS evidence. *Geochim. Cosmochim. Acta* **60**, 3631-3642 (1996).
50. Emerson, S. R. & Huested, S. S. Ocean anoxia and the concentrations of molybdenum and vanadium in seawater. *Mar. Chem*. **34**, 177-196 (1991).
51. Kronberg, B. I. Weathering dynamics and geosphere mixing with reference to the potassium cycle. *Phys. Earth Planet. Inter*. **41**, 125-132 (1985).
52. Meybeck, M. Global chemical weathering of surficial rocks estimated from river dissolved loads. *Am. J. Sci.* **287**, 401-428 (1987).
53. Boyle E. A. Chemical accumulation variations under the Peru Current during the past 130,000 years. *J. Geophys. Res.* **88**, 7667-7680 (1983).
54. Beckmann, B., Flögel, S., Hofmann, P., Schulz, M & Wagner, T. Orbital forcing of Cretaceous river discharge in tropical Africa and ocean response. *Nature* **437**, 241-244 (2005).
55. Singer, A. The paleoclimatic interpretation of clay minerals in soils and weathering profiles. *Earth Sci. Rev.* **15**, 303-326 (1980).
56. Yarincik, K. M., Murray, R. W. & Peterson, L. C. Climatically sensitive eolian and hemipelagic deposition in the Cariaco Basin, Venezuela, over the past 578,000 years: Results from Al/Ti and K/Al. *Paleoceanography* **15**, 210-228 (2000).
57. Santiago Ramos, D. P., Morgan, L. E., Lloyd, N. S. & Higgins, J. A. Reverse weathering in marine sediments and the geochemical cycle of potassium in seawater: Insights from the K isotopic composition ($^{41}K/^{39}K$) of deep-sea pore-fluids. *Geochim. Cosmochim. Acta* **236**, 99-120 (2018).
58. Spears, D. A. & Kanaris-Sotiriou, R. K. Titanium in some carboniferous sediments from great britain. *Geochim. Cosmochim. Acta* **40**, 345-351 (1976).
59. Schmitz, B. The $TiO_2Al_2O_3$ ratio in the Cenozoic Bengal Abyssal Fan sediments and its use as a paleostream energy indicator. *Mar. Geol.* **76**, 195-206 (1987).

60. Shimmield, G. B., Mowbray, S. R. & Weedon, G. P. A 350 ka history of the Indian southwest monsoon - evidence from deep-sea cores, northwest Arabian sea. *Trans. R. Soc. Edinb. Earth Sci.* **81**, 289-299 (1990).

61. Berner, R.A. & Rao, J.-L. Phosphorus in sediments of the Amazon River and estuary: Implications for the global flux of phosphorus to the sea. *Geochim. Cosmochim. Acta* **58**, 2333-2339 (1994).

62. Ruttenberg, K.C. The global phosphorus cycle. *Treatise on geochemistry* **8**, 682 (2003).

63. Ingall, E. D., Bustin, R. M. & Van Cappellen, P. Influence of water column anoxia on the burial and preservation of carbon and phosphorus in marine shales. *Geochim. Cosmochim. Acta* **57**, 303-316 (1993).

64. Van Cappellen, P. & Ingall, E. D. Benthic phosphorus regeneration, net primary production, and ocean anoxia: a model of the coupled marine biogeochemical cycles of carbon and phosphorus. *Paleoceanography* **9**, 677-692 (1994).

65. Slomp, C. P., Epping, E. H., Helder, W. & Raaphorst, W. V. A key role for iron-bound phosphorus in authigenic apatite formation in North Atlantic continental platform sediments. *J. Mar. Res.* **54**, 1179-1205 (1996).

66. Slomp, C. P., Van der Gaast, S. J. & Van Raaphorst, W. Phosphorus binding by poorly crystalline iron oxides in North Sea sediments. *Mar. Geol.* **52**, 55-73 (1996).

67. Bjerrum, C. J. & Canfield, D. E. Ocean productivity before about 1.9 Gyr ago limited by phosphorus adsorption onto iron oxides. *Nature* **417**, 159-162 (2002).

68. Reinhard, C. T. et al. Evolution of the global phosphorus cycle. *Nature* **541**, 386-389 (2017).

69. Ingall, E. & Jahnke, R. Evidence for enhanced phosphorus regeneration from marine sediments overlain by oxygen depleted waters. *Geochim. Cosmochim. Acta* **58**, 2571-2575 (1994).

70. Slomp C. P., Thomson, J. & de Lange G. J. Controls on phosphorus regeneration and burial during formation of eastern Mediterranean sapropels. *Mar. Geol.* **203**, 141–159 (2004).

71. Ruttenberg, K. C. & Berner, R. A. Authigenic apatite formation and burial in sediments from non-upwelling, continental margin environments. *Geochim. Cosmochim. Acta* **57**, 991-1007 (1993).

72. Xiong et al. Phosphorus cycling in Lake Cadagno, Switzerland: A low sulfate euxinic ocean analogue. *Geochim. Cosmochim. Acta* **251**, 116-135 (2019).

73. Ruttenberg, K. C. Development of a sequential extraction method for different forms of phosphorus in marine sediments. *Limnol. Oceanogr.* **37**, 1460-1482 (1992).

74. Thompson, J. et al. Development of a modified SEDEX phosphorus speciation method for ancient rocks and modern iron-rich sediments. *Chem. Geol.* **524**, 383-393 (2019).
75. Canfield, D. E. The geochemistry of river particulates from the continental USA: Major elements. *Geochim. Cosmochim. Acta* **61**, 3349-3365 (1997).
76. Poulton, S. & Raiswell, R. The low-temperature geochemical cycle of iron: from continental fluxes to marine sediment deposition. *Am. J. Sci.* **302**, 774-805 (2002).
77. Poulton, S. W. & Canfield, D. E. Ferruginous conditions: a dominant feature of the ocean through Earth's history. *Elements* **7**, 107-112 (2011).
78. Poulton, S. W. et al. A continental-weathering control on orbitally driven redox-nutrient cycling during Cretaceous Ocean Anoxic Event 2. *Geology* **43**, 963-966 (2015).
79. Pasquier, V., Bryant, R. N., Fike, D. A. & Halevy, I. Strong local, not global, controls on marine pyrite sulfur isotopes. *Sci. Adv.* **7**, eabb7403 (2021).
80. Canfield, D. E., Raiswell, R. & Bottrell, S. The reactivity of sedimentary iron minerals toward sulfide. *Am. J. Sci.* **292**, 659-683 (1992).
81. Poulton, S. W., Krom, M. D. & Raiswell, R. A revised scheme for the reactivity of iron (oxyhydr) oxide minerals towards dissolved sulfide. *Geochim. Cosmochim. Acta* **68**, 3703-3715 (2004).
82. Scholz, F. et al. On the isotope composition of reactive iron in marine sediments: Redox shuttle versus early diagenesis. *Chem. Geol.* **389**, 48-59 (2014).
83. Dal Corso, J. et al. Permo–Triassic boundary carbon and mercury cycling linked to terrestrial ecosystem collapse. *Nat. Commun.* **11**, 1-9 (2020).
84. Sarmiento, J. L. & Toggweiler, J. R. A new model for the role of the oceans in determining atmospheric $P\text{co}_2$. *Nature* **308**, 621-624 (1984).
85. Lenton, T. M., Daines, S. J. & Mills, B. J. W. COPSE reloaded: An improved model of biogeochemical cycling over Phanerozoic time. *Earth-Sci. Rev.* **178**, 1-28 (2018).
86. Walker, J. C. G. & Kasting, J. F. Effects of fuel and forest conservation on future levels of atmospheric carbon dioxide. *Glob. Planet Change* **5**, 151-189 (1992).
87. Rampino, M. R. & Caldeira, K. Major perturbation of ocean chemistry and a 'Strangelove Ocean' after the end-Permian mass extinction. *Terra Nova* **17**, 554-559 (2005).
88. Broecker, W. S. & Peng, T. H. *Tracers in the Sea*. (Columbia University, 1982).
89. Zeebe, R. R. & Wolf-Gladrow, D. *CO_2 in seawater: equilibrium, kinetics, isotopes.* (Elsevier, 2001).

90. Clarkson, M. O. et al. Ocean acidification and the Permo-Triassic mass extinction. *Science* **348**, 229-32 (2015).

91. Lunt, D. J. et al. Earth system sensitivity inferred from Pliocene modelling and data. *Nat. Geosci.* **3**, 60-64 (2010).

92. Mills, B. J. W. et al. Modelling the long-term carbon cycle, atmospheric CO_2, and Earth surface temperature from late Neoproterozoic to present day. *Gondwana Res.* **67**, 172-186 (2019).
