# Supplementary material for: Dynamic redox and nutrient cycling response to climate forcing in the Mesoproterozoic ocean
Source: Nat Commun. 2023 Oct 20;14:6640. doi: 10.1038/s41467-023-41901-7 (PMC10589307; doi:10.1038/s41467-023-41901-7)
Supplement: Supplementary file 3 — Description of Additional Supplementary Files [file 41467_2023_41901_MOESM3_ESM.pdf]

Description of Additional Supplementary Files:

Supplementary Data 1: Analytical data including TOC, Fe speciation, trace element concentrations and pyrite sulfur isotopes. ND = Not determined

Supplementary Data 2: Analytical data including major elements and P phases.
